# A systematic review of social, economic and diplomatic aspects of short-term medical missions

**DOI:** 10.1186/s12913-015-0980-3

**Published:** 2015-09-15

**Authors:** Paul H. Caldron, Ann Impens, Milena Pavlova, Wim Groot

**Affiliations:** 1 Maastricht Graduate School of Governance, University of Maastricht, Maastricht, Netherlands; 2 Midwestern University Institute for Healthcare Innovation, Downers Grove, Illinois USA; 3 Department of Health Services Research; CAPHRI, Maastricht University Medical Center, Faculty of Health, Medicine and Life Sciences, Maastricht University, Maastricht, Netherlands

**Keywords:** Medical missions, Short-term, Professionalization

## Abstract

**Background:**

Short-term medical missions (STMMs) represent a grass-roots form of aid, transferring medical services rather than funds or equipment. The objective of this paper is to review empirical studies on social, economic and diplomatic aspects of STMMs.

**Methods:**

A systematic literature review was conducted by searching PubMed and EBSCOhost for articles published from 1947–2014 about medical missions to lower and middle income countries (LMICs). Publications focused on military, disaster and dental service trips were excluded. A data extraction process was used to identify publications relevant to our objective stated above.

**Results:**

PubMed and EBSCOhost searches provided 4138 and 3262 articles respectively for review. Most articles that provide useful information have appeared in the current millennium and are found in focused surgical journals. Little attention is paid to aspects of volunteerism, altruism and philanthropy related to STMM activity in the literature reviewed (1 article). Evidence of professionalization remains scarce, although elements including guidelines and tactical instructions have been emerging (27 articles). Information on costs (10 articles) and commentary on the relevance of market forces (1 article) are limited. Analyses of spill-over effects, i.e., changing attitudes of physicians or their communities towards aid, and characterizations of STMMs as meaningful foreign aid or strategic diplomacy are few (4 articles).

**Conclusions:**

The literature on key social, economic and diplomatic aspects of STMMs and their consequences is sparse. Guidelines, tactical instructions and attempts at outcome measures are emerging that may better professionalize the otherwise unregulated activity. A broader discussion of these key aspects may lead to improved accountability and intercultural professionalism to accompany medical professionalism in STMM activity.

**Electronic supplementary material:**

The online version of this article (doi:10.1186/s12913-015-0980-3) contains supplementary material, which is available to authorized users.

## Introduction

Few other direct care non-governmental organizations (NGOs) enjoy the level of awareness in western nations as the French-based Médecins Sans Frontières (MSF; Doctors Without Borders) [1]. Through favorable press and fund-raising materials, MSF is recognized primarily for its full time deployment of physicians and support personnel to impoverished areas worldwide where the health of non-combatants suffers by way of conflict, natural disasters, epidemics and famine. Less noticed on a global basis is the activity whereby physicians who are gainfully engaged in medical or surgical practice in their home countries spend short periods away in lower and middle income countries (LMICs^1^), without pay, to provide services directly to the ostensibly poor. Such “missions” are planned and represent a highly direct expression of transnational aid in a novel format. The appellation “short term medical missions” (STMMs) appears to have been informally adopted generally to distinguish these excursions from ad hoc responses to domestic or external disasters, compensated full-time relief practice such as MSF, military and other governmental relief expeditions and residency training programs [2–4]. Authors on STMMs commonly conjecture that the praxis of STMMs has increased over time, although formal evidence of an increase is not readily found [2, 3, 5]. The impact of STMMs on receiving countries’ community health or economics may not yet be measurable [6]. Apart from intended direct therapeutic effects sought for individual patients, STMMs may be viewed from outside as foreign aid with diplomatic ramifications and as a market for unmet needs on both sides of the transaction.

The core competency of medical practice is the reduction of human physical and mental suffering resulting from disease and injury. Physicians in rich countries have ample opportunity to execute these competencies domestically, paid or unpaid. Why do some go abroad pro-bono in the exercise of STMMs? Do the broader consequences of STMMs influence the propensity for physicians to carry out these expeditions?

The objective of this paper is to review empirical studies on the social, economic and diplomatic aspects of STMMs. For this purpose, the method of systematic literature review was adopted. The review focused on studies published from 1947–2014 about STMMs to LMICs.

The review is of relevance not only to research, but also to fostering a more global discourse on social properties accruing to STMMs that run in parallel to the clinical efforts. Observing the praxis of STMMs through the lens of psychosocial, diplomatic and market dynamics may shed light on pathways to maximize its utility and minimize negative externalities as the activity expands.

## Background

Three previous systematic reviews on STMMs have been published [3, 5, 7]. Martiniuk et al. sought “to better understand missions and their potential impact on health systems” in LMICs [3]. Their review identified the most frequent sending and receiving countries as well as dominant types of medical and surgical activities and tabulated the perceived benefits and common criticisms of short term medical missions. Their analysis punctuated the paucity of literature providing quantitative data on prevalence, costs, quality, regulation and outcomes. Shrime et al. compared platforms for elective humanitarian surgery, suggesting that the ad hoc service mission was of value only when self-contained mission vessels, i.e., hospital ships or aircraft, and dedicated local operating facilities were unavailable [7]. More recently, Sykes collated articles about STMMs wherein any attempt was made to collect data on treatment interventions, costs, cost-effectiveness, quality assessment or surveys of perspectives of involved parties. Sykes concluded that reporting has dealt largely with “output rather than outcomes” [5].

These prior reviews have also suggested that the preponderance of published articles on STMMS, numbering in the hundreds, are reflective in nature and convey the emotional rewards, sense of renewal and adventure garnered by physicians in the course of their participation in STMMs [3, 5]. The reviews leave aside the search for theory-based exposés on this form of professional volunteerism as well as commentary on potential spill-over effects. Eckhauser and Freishlag suggest that STMM volunteerism proceeds naturally from the elements of the Hippocratic Oath and serves to restore “personal and professional satisfaction” away from the administrative burden of domestic practice [8]. While acknowledging the body of literature on volunteerism, Withers et al. accepted that individual motives for volunteering are unknown [9].

Taking an alternative viewpoint, we sought an understanding of the social, economic and diplomatic consequences on STMM deployment. How do personal expenditures factor in? Has STMM activity reached such a level of organization that peer influence among licensed professionals, quality measures and cost-effectiveness influence the manner in which they are carried out? We address these questions under the rubric of professionalization, a social process whereby an occupation or trade elevates its integrity and competence through association and scientific advancement, among other steps [10, 11]. Specifically, we refer to professionalization not of the participating physicians, but rather of the praxis of STMMs. Is the activity conceptualized diplomatically as foreign aid or as strategic persuasion? Do STMM participants consider economic or market perspectives such as the market failures that may be addressed through this kind of philanthropy relevant or what it is that they seek via the transaction?

Among the universe of potential aspects, we focused on a limited selection distilled in our five key questions (Table 1) as a framework for meeting our research objective. We chose these questions because they allow us to organize the current literature for commentary on intended or unintended social, economic and diplomatic aspects and their consequences on STMM activity. Thus, the resulting exploratory, qualitative review did not begin from a theory-based perspective nor attempt to generate theory. Rather, we applied a framework synthesis [12] using categories stipulated by the five key questions. We selected three of our key questions because they align conceptually with the three broad, principal reasons suggested by Oxfam^2^ for US foreign aid: national security, economics and applying normative values [13]. The two additional key questions regarding professionalization and costs were selected because they are relevant to the sustainability of STMM activity, so cogent in our time when international commissions discuss the sustainability of development goals and global governance in health at the political level in the post-2015 agenda [14]. We believe these areas merit attention because of the global socio-economic and geopolitical context in which STMM activity occurs.

**Table 1:** Key Questions of the Systematic Review

1	Have STMMs been critically analyzed with respect to normative values of volunteerism, altruism and philanthropy?
2	Have elements of professionalization of STMM activity emerged?
3	Have authors addressed basic economic principles that govern transactions between parties, in this context, between physicians and recipients of care, or other market mechanisms in STMM activity?
4	Are there data on personal costs or cost-effectiveness of STMMs?
5	Spill-over effects: Do physicians view STMMS as foreign aid? Do participating physicians view STMMs as diplomacy or as part of strategic soft power?

## Methods

### Keywords and databases

The construction of the chain of key words and the systematic search in the databases were assisted by a university librarian. Articles were identified from PubMed of the US National Institutes of Health Library of Medicine using key words with MESH terms as the primary search database. A search term algorithm similar to that used by Martiniuk et al. [3] was used after adjusting for dates to include the 64 years from 1947 to 2014. Additional terms were added to facilitate the exclusion of dental and military based mission articles. No requirement for English language was implemented. ESBCOhost was subsequently searched using more limited terms for the same period as the secondary search database (see Search Terms text boxes). Finally, the bibliographies of the articles selected from PubMed and EBSCOhost reviews were searched for additional relevant articles.

### Search Terms:

*PubMed*

**Table:** 

("medical missions, official"[MeSH Terms] OR "medical missions, official"[MeSH Terms]) OR (((("1950/08/01"[PDAT] : "3000"[PDAT]) AND (("brigade"[All Fields] OR (("mission"[All Fields] AND "short-term"[All Fields]) OR ("mission"[All Fields] AND "overseas"[All Fields]) OR ("mission"[All Fields] AND "foreign"[All Fields])) OR (("trip"[All Fields] AND "short-term"[All Fields]) OR ("trip"[All Fields] AND "overseas"[All Fields]) OR ("trip"[All Fields] AND "foreign"[All Fields]))) OR (("mission"[All Fields] AND "volunteer"[All Fields]) OR ("trip"[All Fields] AND "volunteer"[All Fields]) OR ("short-term"[All Fields] AND "volunteer"[All Fields]) OR ("overseas"[All Fields] AND "volunteer"[All Fields]) OR ("foreign"[All Fields] AND "volunteer"[All Fields])) OR (("foreign"[All Fields] AND "humanitarian"[All Fields]) OR ("short-term"[All Fields] AND "humanitarian"[All Fields]) OR ("overseas"[All Fields] AND "humanitarian"[All Fields])) OR ("medical mission"[All Fields] OR "medical missions"[All Fields]) OR (("assistance"[All Fields] AND "short-term"[All Fields]) OR ("assistance"[All Fields] AND "overseas"[All Fields]) OR ("assistance"[All Fields] AND "foreign"[All Fields]) OR ("assistance"[All Fields] AND "humanitarian"[All Fields]) OR ("assistance"[All Fields] AND "volunteer"[All Fields]))) AND "humans"[MeSH Terms]) NOT (("dental clinics"[MeSH Terms] OR ("dental"[All Fields] AND "clinics"[All Fields]) OR "dental clinics"[All Fields] OR "dental"[All Fields]) OR ("dentistry"[MeSH Terms] OR "dentistry"[All Fields]) OR "dent"[All Fields])) NOT ("military personnel"[MeSH Terms] OR "military personnel"[All Fields] OR "military"[All Fields]))	

*EBSCOhost*

**Table:** 

[“medical mission” OR “medical missions” AND “short term”]	

### Screening process

The PRISMA (Preferred Reporting Items for Systematic Reviews and Meta-analyses) guidelines were implemented to standardize the elements of this systematic review [15]. Additional file 1 provides the PRISMA 2009 checklist. Two authors (PC, AI) independently screened the search results and concurred on the final relevant selections. Eligibility of potentially relevant articles was assessed throughout the entire review process to assure that enough detail was provided in the article to determine if inclusion and exclusion criteria were satisfied. For the primary (PubMed) search, the first filter involved review of titles and abstracts for indications that the article was related to STMMs per our above description. The second filter involved review of titles and abstracts that passed the first filter for indications that the article may be relevant to one of the key questions of the systematic review. The third filter involved reviewing the full text of articles passing the first and second filters to determine if the article satisfied each of our inclusion and exclusion criteria and was in fact relevant to a key question. For the secondary (EBSCOhost) search, the same filters were applied to new titles not found in the primary search. Lastly, the bibliographies of the articles selected from the primary and secondary databases were then evaluated with the filtering algorithm applied to the databases.

### Inclusion Criteria

Articles published between 1947 and 2014 were selected if they were available in indexed journals from the databases and if they discussed medical and surgical missions designed to provide direct patient care in LMICs wherein the physician participants are principally gainfully engaged in their home countries. The scope of this study relates to planned and scheduled (non-emergent) missions using unpaid volunteer physicians for short term direct patient care excursions from high income countries to LMICs. Articles were not excluded on the basis of peer review if they provided at least one unique perspective related to a key question. Because the number of full articles focused on our key questions was low, we elected to include editorials, letters or commentaries in the selection process if they otherwise satisfied all inclusion and exclusion criteria, consistent with a framework synthesis.

### Exclusion Criteria

Articles with a primary focus on relief efforts in participants’ home countries, responsive disaster relief or missions that require effective cessation of the physician participants’ home practices were not included in this review. Articles that dealt with medical services by government sector agencies or the military, the logistics of medical supplies, dentistry, nursing, other ancillary services or not related to direct voluntary medical services to patients were also excluded.

### Analysis

The articles included in the final list were thoroughly reviewed and information related to the five questions listed in Table 1 was extracted. Based on this, articles were classified. When articles similarly touched on multiple questions, consensual discretion was used to assign the articles to the categories that appeared most relevant. As articles and studies were primarily qualitative in nature, no summary measures were calculated.

## Results

**Fig. 1:**
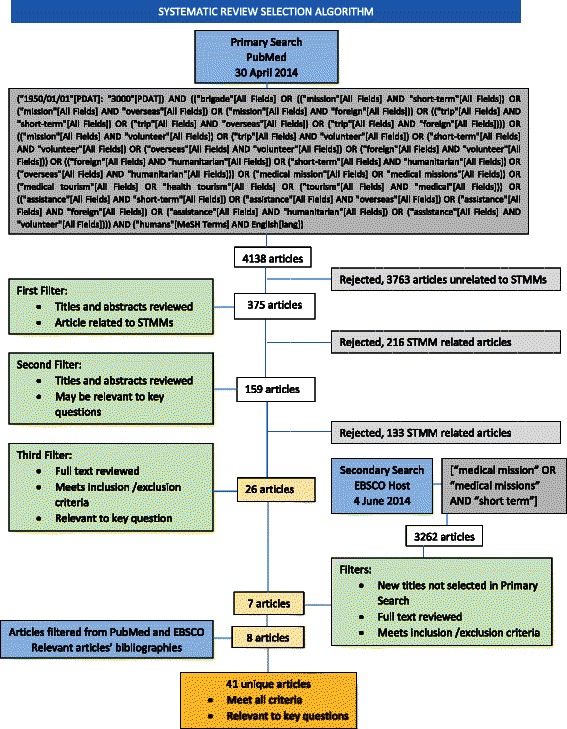
Systematic Review Search and Selection Algorithm

Figure 1 illustrates the search algorithm and results of the filtering process of the primary and secondary database searches and bibliography review of articles selected from the databases on the basis of the inclusion and exclusion criteria. Applying the filters and criteria, composite inter-observer correlation approached 1, with only 10 articles in the search algorithm among more than 4000 initial titles requiring co-deliberation for ultimate selection or rejection. In composite, 41 unique articles were ultimately deemed to meet criteria and provide relevant information on short-term non-compensated medical missions as distributed among the key questions (Table 2). One article (Maki et al., 2008) applied to 3 key questions and one article (Chapin and Doocy, 2010) applied to 2 key questions; the remainder applied to a single key question. Relevant information was extracted from the publications and analyzed as described in the methods section. Characteristics of the publishing journals including country origin, manuscript type, refereed status and discipline base of the selected articles are displayed in Table 2. The key findings of the review are subsequently described.

**Table 2:** Overall description of the articles selected for the review

Relation to the systematic review question areas	Total	Used in prior category(ies)	Unique
1. Volunteerism, altruism and philanthropy	1	-	1
2. Professional professionalization			
a. Professionalization	5	-	5
b. Guidelines	14	1	13
c. Organization – “How To”	9	-	9
3. Analysis of market forces	1	-	1
4. Expenditures for STMMs	10	2	8
5. Spill-over effects	4	-	4
Year of publication			
1. 1947-1980	-		
2. 1981-2000	3		
3. 2001-2005	6		
4. 2006-2010	14		
5. 2011-2014	18		
Journal origin			
1. USA	36		
2. Netherlands	1		
3. Canada	1		
4. International or nationality indeterminate	3		
Type of manuscript			
1. Journal article	37		
2. Editorial or letter	4		
Peer review			
1. Refereed	37		
2. Non-refereed	4		
Discipline of journal			
1. Craniofacial / plastic surgery	12		
2. General surgery	9		
3. General medical	5		
4. Health science/ public health	5		
5. Orthopedics	3		
6. Dermatology	3		
7. Anesthesiology	2		
8. Pediatric medical	1		
9. Economics	1		

### Volunteerism, Altruism and Philanthropy

While it may be considered a *sine qua non* that altruism and philanthropy motivate medical and surgical volunteerism, our systematic review retained a singular article that addressed our key question on volunteerism, altruism [16] or philanthropy in reference to STMMs. In the context of an exhaustive paper designed to orient surgeons to international volunteerism, Pezzella [17] briefly referred to proceedings of an October, 2003 symposium at Trinity College as reported by Alkire and Chen [18] wherein the motivation for unspecified global health initiatives was characterized as deriving from four schools of moral values^3^.

### Professionalization and elements of professionalization

Our review identified no journal wholly dedicated to STMMs. Reports are generally scattered among medical and surgical specialty and subspecialty publications. Further, our online search did not reveal a national or international periodic congress dedicated to the issues and practice of STMMs. The publications in Table 3 convey commentary on or attempts at collectivism beyond a collaborative project, guidelines or set of instructions. Butler published a worldwide online search for any networks for volunteer pediatric surgery and found none [19]. Fisher and Fisher lucidly describe the need, challenges and barriers to collaboration among surgical STMM NGOs [20]. In the enduring competition among NGOs for sources of funding, failure to cooperate with other NGOs and military resources was catalogued by Welling et al. as the third of seven sins of humanitarian medicine [21].

**Table 3:** Professionalization

Author	Journal	Year	Contribution
Maki et al. [2]	*BMC Health Services Research*	2008	Designed instruments to evaluate mission quality
Butler [19]	Journal of Pediatric Surgery	2009	Published Online Search that failed to find collaborative volunteer pediatric surgery network
Kingham [71]	Bulletin of the American College of Surgeons	2011	Noted absence of mission and volunteer credentialing body
Welling et al. [21]	World Journal of Surgery	2012	Argues that effectiveness of missions suffers from lack of collaboration
Fisher and Fisher [20]	Anesthesia and Analgesia	2014	Reviews pitfalls of non-integration of effort and discusses solutions

In contrast, our literature review pointed to online sites that function as “clearing houses” to direct and match physicians to planned missions and mission organizations. Examples include the American College of Surgery’s OperationGivingBack.facs.org [22] and globalpaediatricsurgery.org, the Global Paediatric Surgery Network.

Elements of professionalization are identifiable in our review. Fourteen articles, all from refereed journals, promoted guidelines for the execution for STMMs. The earliest publication by Yeow et al. in 2002 summarized a consensus congress of volunteer cleft palate mission groups from multiple nations and stands out in its international collectivism [23]. Indeed, half of the articles that espouse guidelines for volunteer missions are found in journals of facial plastic and reconstructive specialties.

Table 4 tabulates the sets of guidelines from various sources identified in this review. One finds sophisticated and detailed guidelines, reviewed and approved by multiple specialty societies linked with surgical procedures [24, 25]. Schneider et al. provided check list forms for mission preparation; the content of their guidelines draws from collaboration and co-authorship across specialties [25, 26] in alignment with the VIPS Guidelines^4^. Ethical guidelines for surgical informed consent, surgical photography in the context of STMMs and pediatric craniofacial missions, as well as guidelines for emergency preparedness and response during STMMs and the performance of special multi-staged reconstructive procedures are available [27–31].

**Table 4:** Guidelines (Surgical)

Author	Journal	Year	Contribution
Yeow et al. [23]	J Craniofacial Surgery	2002	International consensus guidelines for cleft missions
Eberlin et al. [24]	Cleft Palate Craniofacial Journal	2008	Quality assurance guidelines based on 20 years’ experience
Hadlock [31]	Archives of Facial and Plastic Surgery	2008	Keys to optimizing outcomes in multistage procedures
Schneider et al. [25, 29]	Plastic and Reconstructive Surgery	2011	Guidelines for plastic surgery in pediatrics (Part I); Ethical Guidelines (Part II)
Politis et al. [26]	Anesthesia and Analgesia	2011	Guidelines for perioperative care in pediatrics (collaborating specialties)
Holt [27, 28]	Archives of Facial and Plastic Surgery	2012	Ethical guidelines for informed consent (I) and case photography (II)
Vyas et al [30]	Plastic and Reconstructive Surgery	2013	Guidelines for surgical emergency preparedness and response
Grimes et al [32]	World Journal of Surgery	2013	General guidelines applicable to any type of surgical mission
Guidelines (Non-surgical)			
Author	Publication	Year	Contribution
Suchdev et al [34]	Ambulatory Pediatrics	2007	Ethical guidelines for sustainable non-surgical pediatric missions
Maki et al [2]	BMC Health Services Research	2008	Developed six instruments to assess mission quality
Chapin & Doocy [33]	World Health & Population	2010	Integrated broad guidelines for all (surgical/non-surgical) missions
DeCamp [35]	HEC Forum	2011	Aligns ethical guidelines for STMMs with those implemented in the conduct of international clinical drug/device trials

Grimes et al. recently proposed more general guidelines for surgeons when going on a volunteer mission [32]. Drawing attention to the burden of surgical need not addressed by typical world health initiatives, their key recommendations emphasized designing trips to focus on actual local needs, locally coordinated training of host country surgeons and support staff, providing the financial assistance to make the training results sustainable and monitoring not only surgical outcomes but also quality of life outcomes.

No similar sets of proposed tactical guidelines directed toward medical (non-surgical) STMMs were found in this systematic review. Following their convenience survey of predominately general medicine and pediatric mission participants, Chapin and Doocy blended suggestions from the existing literature to reiterate broad strategic guidelines for all manner of STMMs [33]. Ethical guiding principles have been proposed for selecting or organizing pediatric care STMMs by Suchdev et al. [34]. DeCamp [35] insightfully juxtaposed these guidelines with similar principles governing clinical drug trials in developing countries [36].

The contribution of Maki et al. [2] has been frequently cited in the relevant literature since its publication in 2008 (58 count by Google Scholar, May 2014). The authors point out the absence of national and international sanctioning organizations for STMMs as well as the absence of standardized quality measurement instruments for missions or mission organizations. Maki and colleagues pooled impressions from several mission goers to develop a set of instruments designed to assess mission quality. In addition, the lead author established a web site as a clearing house for mission organizations and participants to pool their experiences using these six instruments to serve as templates (STMMconnect.com). Despite the prevalence of citations to Maki’s work, we found only a singular application of the Maki instruments [37]. After five years of disuse, the STMMconnect.com website has been taken down as of May 2013 for lack of activity and funding.

Nine articles could be grouped as “How To”, wherein authors codified single or multiple mission experiences into steps and checklists to assist others in planning and executing missions (Table 5). These differ from guideline articles in that they address the pragmatics of mission accomplishment more than concept or propriety. While the selected set varied broadly in the level of formality, it appears likely that each would engage a readership that other sources might miss and provided at least one unique perspective. Our selection excluded mission reports with anecdotal advice.

**Table 5:** “How To”

Author	Journal	Year	Contribution
Norton [43]	Dermatologic Clinics	1999	Planning dermatologic missions
Landau [38]	North Carolina Medical Journal^a^	2001	Detailed instructions on executing primary care missions
Kightlinger [42]	Medical Economics^a^	2003	Self-care for the mission participant
Hoover et al. [46]	Journal of the National Medical Association	2005	Operational preparations for missions to African countries
Hollier et al. [41]	Journal of Craniofacial Surgery	2010	Preparation, implementation
Boyd [40]	Journal of the American Academy of Dermatology	2012	Specific therapeutic suggestions for dermatologic missions
Ramirez-Fort [45]	International Journal of Dermatology	2013	Planning dermatologic missions
Patel et al. [44]	Annals of Plastic Surgery	2013	Sustainable burn missions
Birman & Kolkin [39]	The Journal of Hand Surgery	2013	Planning hand surgery missions

^a^Non-refereed journals

Landau exhaustively parlayed his experience from a general and family practice perspective into a concise and practical treatise for primary care missions anywhere [38]. Other reports cater to burn, hand and craniofacial surgery missions, dermatology and the well-being of the mission-goer [39–45]. Hoover et al. published extensive operational instructions focused on the preparation for missions to African countries in the Journal of the National Medical Association, a periodical that advocates the interests of African American physicians in the US [46].

### Economic and Market Forces

Altruism may be in part a societal response to market failures to provide public goods [47]. In our single retained article for this key question, Mendoza provided an accessible account of how medical missions arise to redress market failures for essential medical services. Using cleft lip/palate interventions in the Philippines as an example, Mendoza contemplated how the interplay of public, private and mission partnerships can operate and still avoid a crowding-out effect to local physicians. Mendoza further discussed the pitfalls that threaten the success of such partnerships in developing or corrupt environments [48].

No articles were found in our search that address other basic market forces that underlie the transactions that take place between individual providers and recipients during STMMS. Such analysis might inform the concerns regarding distortions between cost and benefit related to STMMs [49].

### Expenditures for STMMs

In parallel to Sykes [5], our systematic review revealed no identifiable sources or reports on pooled data for expenditures for short-term medical missions. Casual references to rough estimates of single missions’ and other cost or cost-effectiveness data occur in isolation (Table 6). Estimating an average expense of $50,000 per mission, Maki et al. projected that an annual direct investment in STMMs from the US alone could readily exceed $250 million [2]. Other unsystematically obtained single mission costs and round figures from mission organization expenditures are seen in sparse reports [33, 49, 50].

**Table 6:** Expenditures for Short Term Medical Missions

Author	Journal	Year	Contribution
Propsner [50]	New Jersey Medicine^a^	1998	$147,000 spent on one US facial reconstruction surgical mission to Africa. $28M in free services annually by one organization
Crown [49]	Tennessee Medicine^a^	2005	$40,480 in direct and opportunity cost for 24 persons for five days work
Dupuis [51]	Plastic and Reconstructive Surgery	2006	$78 average cost of operations if local staff utilized (“minimalist approach”)
Wolfberg [52]	The New England Journal of Medicine	2006	Dramatically higher expenditures if full surgical team travels (in contrast to minimalist approach)
Maki et al. [2]	BMC Health Services Research	2008	$50,000 per mission; $250M annually from US
Magee et al. [53]	World Journal of Surgery	2010	Proposal for re-valuing DALYS
Chapin & Doocy [33]	World Health and Population	2010	$22,647 average cost for a medical mission (convenience survey)
Gosselin et al. [55]	World Journal of Surgery	2011	Orthopedic relief mission costs not more than planned mission costs in relation to DALYS
Chen et al. [72]	World Journal of Surgery	2012	Activity based costing for a 5 day orthopedic mission cost effective in relation to DALYS
Moon et al. [54]	World Journal of Surgery	2012	Cleft lip/palate mission cost effective in relation to DALYS

^a^Non-refereed journals

No articles formally tabulated primary or secondary data on physician direct expenditures or opportunity costs. Pointedly, Crown conjectured that with a visiting non-surgical team’s costs “it would be possible to recruit, educate and retain a local doctor, nurse and support staff to man the same clinic for a year” implying that the trade-off for a five-day “feel-good” experience is unjustified [49].

Platform and local staff integration appear to be factors in mission cost. Arguments suggest that a single physician or small teams of surgeons utilizing local nursing and support staff, i.e., a “minimalist” approach, has a cost efficiency per procedure advantage over fully functional travelling teams [51, 52].

Reports are beginning to accumulate wherein activity based costs per disability affected life year saved (DALYS) methodology to assess cost effectiveness is applied [53]. Seminal reports of a one week orthopedic trauma surgery mission and a cleft lip/cleft palate mission asserted their cost effectiveness from both sending and receiving party perspectives [54, 72]. Using DALYS analysis, Gosselin found that cost-effectiveness of trauma surgery in planned STMMs was not favorable compared to disaster-related short-term excursions [55].

### Spill-over effects: Diplomacy, national security and attitudes toward aid

In a chilling tale of perverse consequences in Afghanistan, Fowles’ 1989 editorial punctuated the potent effects that the mere presence of foreign medical teams might have politically [56]. Chiu (non-US) noted that 71.9 % of Taiwanese mission goers saw STMMs as a means to advance Taiwan’s foreign relations [37]. Gorney editorialized incisively on this concept in the context of a facial plastic surgery mission [57]. In describing the role of surgical volunteerism globally, Casey specifically examined survey data and diplomatic literature that supports the concept of medical diplomacy in the domain of counter-terrorism and evolving American security strategy [22] (Table 7). With the exception of Chiu’s survey results from Taiwanese volunteers, other direct connections between diplomatic concerns and STMMS are not forthcoming from the literature.

**Table 7:** Spill-over Effects – National Security

Author	Journal	Year	Contribution
Fowles [56]	Orthopedic Review	1989	Cautionary report on geopolitical ramifications of STMMs
Gorney [57]	Plastic and Reconstructive Surgery	2005	Recognized STMM activity as a potential element of diplomacy
Casey et al. [22]	Surgical Clinics of North America	2007	Attitudes in LMICs towards US improved by medical aid
Chiu [37]	Evaluation and the Health Professions	2012	Prevalent foreign relations motivation in Taiwanese STMM participants

We discovered no articles that explicitly link STMMs to effects on the attitudes of physicians, their families or communities toward any kind of foreign aid or that focus on STMMs as meaningful foreign aid. Commentary on changes in attitudes was confined to other dimensions. For example, Van Tilburg reported a large majority of mission goers responding to a survey felt that their mission experience “broadened their views of the world” and would be willing to repeat the activity [58]. Campbell et al. discuss their own and other published survey results of surgical residents-in-training regarding the value of an overseas experience in the development of cross-cultural competency [59]. Results of a similar small survey published by Aziz et al. of trainees on cleft missions indicated an increase in “awareness of global healthcare” [60].

## Discussion

Martiniuk et al. identified the USA as the largest among the four leading sending countries for STMMs, ahead of Canada, United Kingdom and Australia [3]. We extended our literature retrospective beyond the reviews of Martiniuk (25 years) and Sykes (20 years) with the objective of capturing any existing seminal publications from near the origins of US hegemony and subsequent trends in the praxis. Whereas Kickbusch pointed to the need to examine US hegemony in global public health in 2002 [61], we found no indication from literature that US hegemony is correlated with the greater representation of US physicians in STMMs. Our review does indicate that the most impactful articles of the last 64 years on guidelines, and hence on the effort to improve quality of volunteer short-term medical missions, have been published in the current millennium, preceded almost exclusively by descriptive articles of a reflective nature. This has coincided with the increase in attention to, scrutiny of and physician participation in STMMs. While most published articles are rejected from this and previous systematic reviews for lack of objective or quantitative contribution, the hundreds of articles regarding STMMs that constitute reflections or productivity reports in print, whether in high or low impact, refereed or non-refereed journals, indicate a palpable affinity among editors and medical readership concerning this activity [3, 5]. The attribution that these missions are charitable and altruistic and thereby valid in their own right may interfere with the inclination for data collection and objective analysis [62].

It is notable therefore that professionalization remains limited. As industries, movements and enterprises rise and eventually obsolesce, one measure of viability and vitality is the evidence of guilds or associations. Participants in professions and stable industries cooperate in such collectives formally to share new ideas, scientific advancements and formulate principles and credentialing by means of repeating focused congresses and journals for the benefit of both participants and society [63]. STMM participation is indeed not a remunerative activity. The licensed professionals who participate may already endure substantial national, local and specialty regulation in addition to peer pressure to join, participate in the congresses of and pay dues to perhaps multiple organizations. We therefore intuit that the non-monetary rewards sought and accumulated through STMM activity may be made even more attractive because of the absence from layers of regulation, credentialing and formality.

Most detailed reporting on mission collaborations, issuance of guidelines and leanings towards professionalization come from surgical literature and surgical organizations. Operation Giving Back [22] with its publication, the Bulletin of the American College of Surgeons (ACS), may be the most proximate example in the US to a burgeoning STMM guild, even though we found no evidence of any recurring congress or membership specifically related to the initiative. Similarly, Volunteers in Plastic Surgery (VIPS), through the affiliation of six major related STMM NGOs, have collaborated on guidelines as noted^5^. No discernable evidence of a correlative centralization of effort in family medicine, internal medicine and pediatrics is found. An appealing explanation for the disparity between surgical and non-surgical cohesiveness trends may be that surgical missions tend to be limited to a finite set of discrete procedures within the scope of more focused skill sets and are more amenable to overall process and outcome assessments. Congruently, the developing world’s burden of surgical disease is less amenable to global public health measures and policy remedies than non-surgical disorders and thus summons the direct relief that surgical STMMs can provide. The direct costs and opportunity costs of surgical missions may be generally more sizable than non-surgical missions and a higher potential for serious adverse events may warrant greater circumspection.

Crown argues with clarity that the “feel good” reward accruing to mission-goers hardly justifies the itemized and total costs and that the funds are misapplied [49]. Such a position overlooks the realpolitik that most such expenditures are discretionary to the mission-goer. This “feel good” sentiment may equate with the concept of “warm glow” popularized in current theory on philanthropy [47, 64]. In the context of the face-to-face interactions occurring in pro-bono medical missions, the intangible reward system may indeed be layers more complex, more habit-forming and compelling than other forms of philanthropy and begs to be better understood. Indeed, marketers of products and services as well as sending and receiving country mission organizers recognize the monetized value of these experiences and emotions and will almost certainly expand ways to exploit them. In this milieu, the first key question of this review regarding normative values should be as rigorously analyzed as outcomes measures, accounting and impact of STMMS since this may be where the disconnect between the clinical practice of STMMs and the due diligence is rooted. Ultimately, the execution of STMMs is critically dependent on the motivation of and decision by physicians to participate in them among other domestic opportunities for physician volunteerism and philanthropy. This issue remains vague and not elucidated by the results of any of the five key questions of this literature review. More prospective analysis of the influence of these aspects on participation would be warranted.

In an era of asymmetrical warfare, western strategic defense is migrating from emphasis on conventional expeditionary might towards counter-terrorism and counter-insurgency supported by robust intelligence and diplomatic soft power [65]. It is implied that pre-empting conflict will depend on skilled diplomacy and influence linked to managing perceptions of the West among peoples of developing countries where unrest may erupt. Nye points out in *The Future of Power* that the sources of soft power wherein the elements of attraction and persuasion lie are socially constructed [66]. McInnes has skillfully interwoven the concepts of international healthcare strategies with modern concepts of soft power [67]. The Cuban physician diaspora, while controversial, may be the most acknowledged example of a nation providing direct medical assistance as a lever of diplomacy, though it is not voluntary on the part of the physician, nor short term nor uncompensated [68]. Done well, STMMs may have the makings of an effective civil outreach as one fiber of western ground-level diplomacy to undermine popular support for terror and insurgent groups.

### Bias and limitations

Bias may be expected in several forms beginning with our selection of relevant articles. The limitation of the selection to medical and surgical mission focus may omit some closely related input of value from ancillary activities such as dentistry, nursing and medical education. Most articles on STMMs are qualitative in nature and thus bias is inherent; such bias may carry over into this review. Selected articles originate almost exclusively from authorship within a high-income sending country such that bias may arise from a rich country set of values.

A manifest limitation in our analysis is the relatively few articles that satisfied our criteria. The usual danger of such a review as ours is that the absence of evidence would be misconstrued as evidence of absence of a particular effect. The actual impact of these social, economic and diplomatic aspects on the decision of physicians to participate in STMMs should be assessed prospectively through well designed surveys beyond the published retrospective surveys.

## Conclusions

Our objective to assess certain social, economic and diplomatic aspects of STMMs reveals little attention from the literature we reviewed. Scant analyses exist in the current literature wherein normative concepts of volunteerism, altruism and philanthropy is applied specifically to the praxis of STMMs. Transactional analysis and study of other market dynamics is wont; the enlightened self-interest of the parties in the exchange, a universal motivator, is not openly explored. Estimations and commentary over related expenditures are found, but accounting of direct physician outlays and how such costs may affect the decision to go is yet unattended. With the possible exception of Taiwanese missions, diplomacy, foreign aid and projection of soft power do not appear to be expressed determinants of participation. Professionalization appears underdeveloped, though elements of professionalization such as guidelines, codified instructions and implementation of cost benefit analysis are accumulating.

### Policy and Practice Implications

STMMs may perform a function of providing needed services where markets have failed. As rich and poor economies converge, the inclination for this and other forms of aid should obsolesce. In the meantime, it is likely that the call for value, meaning a rational return on investment measured by dollars per DALYS or similar unit, will continue in the medical and social science literature and media [3, 21]. Mitigation of potential externalities such as crowding-out local health care provision and adverse medical events related to inadequate follow-up, among others, is another target. As western security policy evolves, the social perception elements of soft power may render cross-cultural acumen on the part of participating physicians and their support teams an inescapable requirement. Such improvements need not diminish the “warm glow” so precious to the providers and embedded in the answer to why they go. Achieving accountability and cross-cultural skills commensurate with the medical professionalism displayed in STMMs may better advance from greater interchange among disparate actors. Peer pressure and peer acknowledgment as motivators compounded through professionalization would also be unlikely to dim the glow. Formation of a national or international organization specifically focused on STMMs with a dedicated journal and repeating congresses need not add to the regulatory burden on physicians and could serve as a platform for quality enhancement.

An economist would reasonably ask if the composite expenditures on STMMs worldwide are material, then conclude from the lack of data and sources of data, as well as the inattention to the regulation of the activity, that it is not. Nonetheless, there exists at least the impression that this socioeconomic activity is expanding and the cumulative expenses including expenditures and opportunity costs may be consequential. As aid, Martiniuk et al. amply characterize this transfer of skilled labor as opposed to cash transfers in their title “Brain Gains…” Changing the present US tax code that provides indirect support of STMMs by allowing deduction of all applicable costs from taxable income could have a potential dampening effect on participation. A broader consideration of STMMs as a grassroots expression of foreign aid and a potential instrument of security policy may therein be justified.

As non-state actors, volunteer physicians’ professional acumen in the concepts of effective foreign aid and promotion of peace could benefit from a collaborative approach. Educational interaction with actors in diplomatic and security sectors could bring further value to STMM activity. Facilitating a broader awareness of the role of the STMM among overall foreign aid objectives could benefit similarly. Absent these perspectives, the unattractive consequences that sometimes adversely stigmatize STMM activity may expand in similar dimension to growth of the activity itself.

### Future research implications

Neither this nor previous reviews profile “who” goes on uncompensated medical service trips; that may hold much insight into “why”. Further perspectives from care-receiving patients and communities beyond the paucity of on-site surveys available are needed to refine mission sophistication and prescription [2, 70]. Quantification and impact of personal costs on physician participation should be explored. Recipient communities’ attitudes towards “the West” as well as western attitudes towards service aid are potential positive externalities of STMMs that could be better informed through exchange with the diplomatic corp.

## Additional file

Additional file 1: **PRISMA 2009 Checklist.** (DOC 63 kb)

## Abbreviations

STMM: Short-term medical mission
LMIC: Lower and middle income country
NGO: Non-governmental organization
MSF: Médecins sans Frontières
DALYS: Disability affected life year saved
ACS: American College of Surgeons
VIPS: Volunteers in Plastic Surgery

## Footnotes

1 World Bank list of economies, February 2014, siteresources.worldbank.org/DATASTATISTICS/Resources/CLASS.XLS.

2 Oxfam is an international confederation of 17 organizations working in approximately 94 countries worldwide to find solutions to poverty and what it considers injustice around the world. https://www.oxfam.org/en/about/ Accessed 29 July 2015.

3 Humanitarianism, acting virtuously towards those in need, based on compassion, empathy, or altruism as mandated in the philosophy of great religions; utilitarianism, maximizing aggregate subjective happiness, since general good health is in the best interest of all including those not inclined to altruism; equity, a fairer distribution of health capabilities; rights - fulfilling our obligations so others are dignified [18].

4 Plastic Surgery Education Foundation: Volunteers in Plastic Surgery (VIPS) Guidelines for the Care of Children in the Less Developed World. Version 5.0. January 15, 2009.

5 Austin Smiles, Rotaplast, ReSurge International, Nicaplast, Operation Smile, Small World Foundation.
